# Supplementary material for: A flagellate-to-amoeboid switch in the closest living relatives of animals
Source: eLife. 2021 Jan 15;10:e61037. doi: 10.7554/eLife.61037 (PMC7895527; doi:10.7554/eLife.61037)
Supplement: Supplementary file 1. [file elife-61037-supp1.docx]

**Supplementary File 1. Cell crawling across animal diversity.**

| **Phylum** | **Crawling cell types in adult organisms** | **Crawling cells during development** | **Crawling cells during wound healing** |
| --- | --- | --- | --- |
| **Porifera** | Archeocytes^1^ (absent in calcaroneans^2^)  Amoeboid sperm in *Leucosolenia*^3^ | Transiently amoeboid oocytes^4,5^  Ingression during larval development in some species^6^ | Epithelial-to-mesenchymal transition^7^ or epithelial migration^8^ of wounded pinacocytes  Crawling of mesohyl cells toward the wound^8^ |
| **Ctenophora** | Phagocytic stellate cells^9^ | Transiently amoeboid oocytes^10^ | Migration of mesogleal cells toward the wound^11^ |
| **Placozoa** | *None reported* | *Post-cleavage development unknown*  Possible oocyte formation from epithelial cells by epithelial-to-mesenchymal transition and cell migration^12,13^ | *Not studied at the cell level* (but a tissue-level study of healing suggests that epithelial re-sealing could occur without active changes in cell behavior^14^) |
| **Cnidaria** | Amoebocytes^15^ | Transiently amoeboid oocytes^16–18^  Primordial germ cell migration^19^  Gastrulation by ingression in some species^20,21^ | Epithelial cell migration^22–24^  Migration of mesogleal cells toward the wound^25^ |
| **Xenacoelomorpha** | *None reported*^26^ | *None reported* | Epithelial-to-mesenchymal transition of wounded epidermal cells^27^ |
| **Echinodermata** | Coelomocytes^28^ | Primordial germ cell migration^29^ | Mesenchymal cells migration toward site of injury^30^ |
| **Hemichordata** | Coelomocytes^31^ | *None reported* (germline development unknown) | Migration toward site of injury^30,32,33^ |
| **Chordata** | Amoebocytes in cephalochordates and urochordates^31,34^  Leukocytes and mesenchymal cells in vertebrates^35^ | Primordial germ cell migration^36–38^  Gastrulation by ingression in some species^39^  Neural crest cells in vertebrates | Epithelial-to-mesenchymal transition in injured epithelia^40^  Leukocyte migration toward site of injury^41–43^ |
| **Arthropoda** | Hemocytes ^44^ | Primordial germ cell migration ^45^  Gastrulation by ingression in some species^46^  Mesenchymal migration of mesodermal cells^47,48^ | Epithelial cell migration in injured epithelia ^49^ |
| **Tardigrada** | *None reported* (so-called brain “amoebocytes” are likely not motile^50^) | Primordial germ cell migration^51,52^  Migration of endodermal and mesodermal precursors^51,52^ | *Not studied* |
| **Onychophora** | Hemocytes^53^ | Primordial germ cell migration^54^  Migration of endodermal and mesodermal precursors^54^ | *Not studied* |
| **Nematomorpha** | *None reported* | *None reported (*embryonic development is little known) | *Not studied* |

| **Phylum** | **Crawling cell types in adult organisms** | **Crawling cells during development** | **Crawling cells during wound healing** |
| --- | --- | --- | --- |
| **Nematoda** | Amoeboid sperm cells using a non-actin-based mechanism^55^ | Actin-based migration of gonad distal tip cells^56^  Migration of head mesodermal cells and male linker cell^57^ | Epithelial cell migration in embryos^58^ but not adults^59^ |
| **Priapulida** | Amoebocytes^60,61^ | *None reported* | *Not studied* |
| **Kinorhyncha** | Amoebocytes^60,62,63^ | *None reported* | *Not studied* |
| **Loricifera** | Coelomocytes^64^ | *None reported* | *Not studied* |
| **Annelida** | Coelomocytes^65^ and hemocytes^44^ | Primordial germ cell migration^66^ | Migration during regeneration^67^ |
| **Platyhelminthes** | *None reported* | *None reported* | Neoblasts migrate to the wounded area upon injury^68^ |
| **Nemertea** | Hemocytes^69^ | Transiently amoeboid oocytes^70^  Migratory proboscis precursors^71^ | Migration during regeneration^72^ |
| **Ectoprocta** | Amoebocytes^73^ | Primordial germ cell migration^74^ | *Not studied* |
| **Phoronida** | Amoebocytes^75,76^ | Migration of mesodermal cells^77,78^ | *Not studied* |
| **Brachiopoda** | Amoebocytes^79,80^ | *None reported* | *Not studied* |
| **Mollusca** | Hemocytes^44,81^ | Transiently amoeboid oocytes^82–85^  Presumptive germ cell migration^86^  Ectomesoderm migration^87^ | Hemocyte migration toward site of injury^88^ |
| **Entoprocta** | *None reported* | *None reported* | *Not studied* |
| **Gastrotricha** | *None reported* | *None reported* | *Not studied* |
| **Gnathostomulida** | *None reported* | *None reported* | *Not studied* |
| **Rotifera** | *None reported* | *None reported* | *Not studied* |
| **Micrognathozoa** | *None reported* | *None reported* | *Not studied* |
| **Chaetognatha** | Amoebocytes^89^ | Primordial germ cells migration^90^ | Epithelial cell migration^91^ |

**Suppelementary Table 1 – References**

1. Brusca, R. C. & Brusca, G. J. *Invertebrates*. (Sinauer Associates, Inc., 2003).

2. Adamska, M. Sponges as models to study emergence of complex animals. *Current Opinion in Genetics and Development* (2016). doi:10.1016/j.gde.2016.05.026

3. Anakina, R. P. & Drozdov, A. L. Gamete Structure and Fertilization in the Barents Sea Sponge *Leucosolenia complicata*. *Russ. J. Mar. Biol.* (2001). doi:10.1023/A:1016761317637

4. Franzen, W. Oogenesis and larval development of *Scypha ciliata* (Porifera, Calcarea). *Zoomorphology* **107**, 349–357 (1988).

5. Haeckel, E. *The evolution of man: a popular exposition of the principal points of human ontogeny and phylogeny*. (1874). doi:10.5962/bhl.title.61275

6. Ereskovsky, A. V. *The comparative embryology of sponges*. *The Comparative Embryology of Sponges* (Springer, 2010). doi:10.1007/978-90-481-8575-7

7. Borisenko, I. E., Adamska, M., Tokina, D. B. & Ereskovsky, A. V. Transdifferentiation is a driving force of regeneration in *Halisarca dujardini* (Demospongiae, Porifera). *PeerJ* (2015). doi:10.7717/peerj.1211

8. Lavrov, A. I., Bolshakov, F. V., Tokina, D. B. & Ereskovsky, A. V. Sewing up the wounds : The epithelial morphogenesis as a central mechanism of calcaronean sponge regeneration. *J. Exp. Zool. Part B Mol. Dev. Evol.* (2018). doi:10.1002/jez.b.22830

9. Traylor-Knowles, N., Vandepas, L. E. & Browne, W. E. Still Enigmatic: Innate Immunity in the Ctenophore *Mnemiopsis leidyi*. in *Integrative and Comparative Biology* (2019). doi:10.1093/icb/icz116

10. Dunlap-Pianka, H. Ctenophora. in *Reproduction of Marine Invertebrates, volume I. Acoelomate and Pseudocoelomate Metazoans* (eds. Giese, A. C. & Pearse, J. S.) (Academic Press, 1974). doi:10.1016/b978-0-12-282501-9.50009-0

11. Ramon-Mateu, J., Ellison, S. T., Angelini, T. E. & Martindale, M. Q. Regeneration in the ctenophore *Mnemiopsis leidyi* occurs in the absence of a blastema, requires cell division, and is temporally separable from wound healing. *BMC Biol.* (2019). doi:10.1186/s12915-019-0695-8

12. Grell, K. G. & Ruthmann, A. Placozoa. in *Microscopic Anatomy of Invertebrates. Volume 2: Placozoa, Porifera, Cnidaria, and Ctenophora* 13–28 (Wiley-Liss, Inc., 1991).

13. Grell, K. G. & Benwitz, G. Elektronenmikroskopische beobachtungen über das wachstum der eizelle und die bildung der ‘befruchtungsmembran’ von *Trichoplax adhaerens* F. E. Schulze (Placozoa). *Zeitschrift für Morphol. der Tiere* (1974). doi:10.1007/BF00277511

14. Prakash, V. N., Bull, M. S. & Prakash, M. Motility induced fracture reveals a ductile to brittle crossover in the epithelial tissues of a simple animal. *bioRxiv* (2019). doi:10.1101/676866

15. Gold, D. A. & Jacobs, D. K. Stem cell dynamics in Cnidaria: Are there unifying principles? *Dev. Genes Evol.* (2013). doi:10.1007/s00427-012-0429-1

16. Honegger, T. G., Zürrer, D. & Tardent, P. Oogenesis in *Hydra carnea*: A new model based on light and electron microscopic analyses of oocyte and nurse cell differentiation. *Tissue Cell* (1989). doi:10.1016/0040-8166 (89)90052-9

17. Larkman, A. U. An ultrastructural study of oocyte growth within the endoderm and entry into the mesoglea in *Actinia fragacea* (Cnidaria, anthozoa). *J. Morphol.* (1983). doi:10.1002/jmor.1051780207

18. Eckelbarger, K. J., Hand, C. & Uhlinger, K. R. Ultrastructural features of the trophonema and oogenesis in the starlet sea anemone, *Nematostella vectensis* (Edwardsiidae). *Invertebr. Biol.* (2008). doi:10.1111/j.1744-7410.2008.00146.x

19. Chen, C.-Y., McKinney, S. A., Ellington, L. R. & Gibson, M. C. Hedgehog signaling is required for endomesodermal patterning and germ cell development in *Nematostella vectensis*. *bioRxiv* (2020). doi:10.1101/2020.01.15.907238

20. Kraus, Y., Chevalier, S. & Houliston, E. Cell shape changes during larval body plan development in *Clytia hemisphaerica*. *bioRxiv* (2019). doi:10.1101/864223

21. Martindale, M. Q. & Byrum, C. A. Gastrulation in the Cnidaria and Ctenophora. in *Gastrulation* (ed. Stern, C. D.) 731 (Cold Spring Harbor Laboratory Press, 2004).

22. Malamy, J. E. & Shribak, M. An orientation-independent DIC microscope allows high resolution imaging of epithelial cell migration and wound healing in a cnidarian model. *J. Microsc.* (2018). doi:10.1111/jmi.12682

23. Kamran, Z. *et al.* In vivo imaging of epithelial wound healing in the cnidarian *Clytia hemisphaerica* demonstrates early evolution of purse string and cell crawling closure mechanisms. *BMC Dev. Biol.* (2017). doi:10.1186/s12861-017-0160-2

24. DuBuc, T. Q., Traylor-Knowles, N. & Martindale, M. Q. Initiating a regenerative response; cellular and molecular features of wound healing in the cnidarian *Nematostella vectensis*. *BMC Biol.* (2014). doi:10.1186/1741-7007-12-24

25. Patterson, M. J. & Landolt, M. L. Cellular reaction to injury in the anthozoan *Anthopleura elegantissima*. *J. Invertebr. Pathol.* (1979). doi:10.1016/0022-2011 (79)90152-6

26. Chiodin, M. *et al.* Mesodermal Gene Expression in the Acoel Isodiametra pulchra Indicates a Low Number of Mesodermal Cell Types and the Endomesodermal Origin of the Gonads. *PLoS One* (2013). doi:10.1371/journal.pone.0055499

27. Geddes, P. Observations on the Physiology and Histology of Convoluta Schultzii. *Proc. R. Soc. London* **28**, 449–457 (1879).

28. Chia, F. S. & Xing, J. Echinoderm coelomocytes. *Zoological Studies* (1996).

29. Campanale, J. P. *et al.* Migration of sea urchin primordial germ cells. *Dev. Dyn.* (2014). doi:10.1002/dvdy.24133

30. Rychel, A. L. & Swalla, B. J. Regeneration in hemichordates and echinoderms. in *Stem Cells in Marine Organisms* (2009). doi:10.1007/978-90-481-2767-2_10

31. Rowley, A. F., Rhodes, C. P. & Ratcliffe, N. A. Protochordate leucocytes: a review. *Zool. J. Linn. Soc.* (1984). doi:10.1111/j.1096-3642.1984.tb01978.x

32. Rychel, A. L. & Swalla, B. J. Anterior regeneration in the hemichordate Ptychodera flava. *Dev. Dyn.* (2008). doi:10.1002/dvdy.21747

33. Rao, K. P. Morphogenesis during regeneration is an enteropneust. *J. Anim. Morphol. Physiol. India* 1–7 (1955).

34. Muñoz-Chápuli, R. & Pérez-Pomares, J. A. M. Origin of the Vertebrate Endothelial Cell Lineage. Ontogeny and Phylogeny. in *Heart Development and Regeneration* (2010). doi:10.1016/B978-0-12-381332-9.00022-0

35. Kierszenbaum, A. L. & Tres, L. L. Blood and hematopoiesis. in *Histology and Cell Biology: An Introduction to Pathology* 752 (Saunders, 2015). doi:10.1016/b978-0-323-07842-9.50010-1

36. Grimaldi, C. & Raz, E. Germ cell migration—Evolutionary issues and current understanding. *Seminars in Cell and Developmental Biology* (2020). doi:10.1016/j.semcdb.2019.11.015

37. Tarbashevich, K. & Raz, E. The nuts and bolts of germ-cell migration. *Current Opinion in Cell Biology* (2010). doi:10.1016/j.ceb.2010.09.005

38. Nieuwkoop, P. D. & Sutasurya, L. A. *Primordial germ cells in the chordates : embryogenesis and phylogenesis*. *Developmental and cell biology series* (Cambridge University Press, 1979).

39. Shook, D. R. & Keller, R. Epithelial type, ingression, blastopore architecture and the evolution of chordate mesoderm morphogenesis. *Journal of Experimental Zoology Part B: Molecular and Developmental Evolution* (2008). doi:10.1002/jez.b.21198

40. Stone, R. C. *et al.* Epithelial-mesenchymal transition in tissue repair and fibrosis. *Cell and Tissue Research* (2016). doi:10.1007/s00441-016-2464-0

41. Powell, D. *et al.* Chemokine Signaling and the Regulation of Bidirectional Leukocyte Migration in Interstitial Tissues. *Cell Rep.* (2017). doi:10.1016/j.celrep.2017.04.078

42. De Oliveira, S., Rosowski, E. E. & Huttenlocher, A. Neutrophil migration in infection and wound repair: Going forward in reverse. *Nature Reviews Immunology* (2016). doi:10.1038/nri.2016.49

43. Sieger, D., Moritz, C., Ziegenhals, T., Prykhozhij, S. & Peri, F. Long-Range Ca2+ Waves Transmit Brain-Damage Signals to Microglia. *Dev. Cell* (2012). doi:10.1016/j.devcel.2012.04.012

44. Stang-Voss, C. On the Ultrastructure of Invertebrate Hemocytes: An Interpretation of their Role in Comparative Hematology. in *Contemporary Topics in Immunobiology, volume 4: Invertebrate Immunology* (ed. Cooper, E. L.) 65–76 (Plenum Press, New York, 1974). doi:10.1007/978-1-4684-3048-6_7

45. Warrior, R. Primordial germ cell migration and the assembly of the *Drosophila* embryonic gonad. *Dev. Biol.* (1994). doi:10.1006/dbio.1994.1306

46. Urbansky, S., Avalos, P. G., Wosch, M. & Lemke, S. Folded gastrulation and T48 drive the evolution of coordinated mesoderm internalization in flies. *Elife* (2016). doi:10.7554/eLife.18318.001

47. Smallhorn, M., Murray, M. J. & Saint, R. The epithelial-mesenchymal transition of the *Drosophila* mesoderm requires the Rho GTP exchange factor pebble. *Development* (2004). doi:10.1242/dev.01150

48. Schäfer, G., Narasimha, M., Vogelsang, E. & Leptin, M. Cadherin switching during the formation and differentiation of the *Drosophila* mesoderm - implications for epithelial-tomesenchymal transitions. *J. Cell Sci.* (2014). doi:10.1242/jcs.139485

49. Wu, Y. *et al.* A Blood-Borne PDGF/VEGF-like Ligand Initiates Wound-Induced Epidermal Cell Migration in *Drosophila* Larvae. *Curr. Biol.* (2009). doi:10.1016/j.cub.2009.07.019

50. Persson, D. K., Halberg, K. A., Jørgensen, A., Møbjerg, N. & Kristensen, R. M. Brain anatomy of the marine tardigrade *Actinarctus doryphorus* (Arthrotardigrada). *J. Morphol.* (2014). doi:10.1002/jmor.20207

51. Hejnol, A. & Schnabel, R. The eutardigrade *Thulinia stephaniae* has an indeterminate development and the potential to regulate early blastomere ablations. *Development* (2005). doi:10.1242/dev.01701

52. Hejnol, A. & Schnabel, R. What a couple of dimensions can do for you: Comparative developmental studies using 4D microscopy - Examples from tardigrade development. *Integr. Comp. Biol.* (2006). doi:10.1093/icb/icj012

53. Silva, J. R. M. C., Coelho, M. P. D. & Nogueira, M. I. Induced inflammatory process in Peripatus acacioi Marcus et Marcus (Onychophora). *J. Invertebr. Pathol.* (2000). doi:10.1006/jipa.1999.4898

54. Anderson, D. T. *Embryology and Phylogeny in Annelids and Arthropods.* (Pergamon Press, 1973). doi:10.2307/2412250

55. Roberts, T. M. & Stewart, M. Nematode sperm: Amoeboid movement without actin. *Trends in Cell Biology* (1997). doi:10.1016/S0962-8924 (97)01113-6

56. Cram, E. J., Shang, H. & Schwarzbauer, J. E. A systematic RNA interference screen reveals a cell migration gene network in *C. elegans*. *J. Cell Sci.* (2006). doi:10.1242/jcs.03274

57. Hedgecock, E. M., Culotti, J. G. & Hall, D. H. The unc-5, unc-6, and unc-40 genes guide circumferential migrations of pioneer axons and mesodermal cells on the epidermis in *C. elegans*. *Neuron* (1990). doi:10.1016/0896-6273 (90)90444-K

58. Raich, W. B., Agbunag, C. & Hardin, J. Rapid epithelial-sheet sealing in the *Caenorhabditis elegans* embryo requires cadherin-dependent filopodial priming. *Curr. Biol.* (1999). doi:10.1016/S0960-9822 (00)80015-9

59. Xu, S. & Chisholm, A. D. A Gαq-Ca2+ signaling pathway promotes actin-mediated epidermal wound closure in *C. elegans*. *Curr. Biol.* (2011). doi:10.1016/j.cub.2011.10.050

60. Schmidt-Rhaesa, A. *The Evolution of Organ Systems*. *The Evolution of Organ Systems* (2007). doi:10.1093/acprof:oso/9780198566687.001.0001

61. McLean, N. Amoebocytes in the Lining of the Body Cavity and Mesenteries of *Priapulus caudatus* (Priapulida). *Acta Zool.* (1984). doi:10.1111/j.1463-6395.1984.tb00811.x

62. Kristensen, R. M. & Hay‐Schmidt, A. The Protonephridia of the Arctic Kinorhynch *Echinoderes aquilonius* (Cyclorhagida, Echinoderidae). *Acta Zool.* (1989). doi:10.1111/j.1463-6395.1989.tb01048.x

63. Neuhaus, B. & Higgins, R. P. Ultrastructure, biology, and phylogenetic relationships of Kinorhyncha. in *Integrative and Comparative Biology* (2002). doi:10.1093/icb/42.3.619

64. Kristensen, R. M. Loricifera. in *Microscopic Anatomy of Invertebrates. Volume 4: Aschelminthes* 448 (Wiley-Liss, Inc., 1991).

65. Baskin, D. G. The Coelomocytes of Nereid Polychaetes. in *Contemporary Topics in Immunobiology, volume 4: Invertebrate Immunology* (ed. Cooper, E. L.) 55–64 (Plenum Press, New York, 1974). doi:10.1007/978-1-4684-3048-6_6

66. Rebscher, N., Zelada-González, F., Banisch, T. U., Raible, F. & Arendt, D. Vasa unveils a common origin of germ cells and of somatic stem cells from the posterior growth zone in the polychaete *Platynereis dumerilii*. *Dev. Biol.* (2007). doi:10.1016/j.ydbio.2007.03.521

67. Zattara, E. E., Turlington, K. W. & Bely, A. E. Long-term time-lapse live imaging reveals extensive cell migration during annelid regeneration. *BMC Dev. Biol.* (2016). doi:10.1186/s12861-016-0104-2

68. Palmberg, I. Cell migration and differentiation during wound healing and regeneration in *Microstomum lineare* (Turbellaria). in *Advances in the Biology of Turbellarians and Related Platyhelminthes* (1986). doi:10.1007/978-94-009-4810-5_25

69. Valembois, P., Roch, P. & Boiledieu, D. Cellular Defense Systems of the Platyhelminthes, Nemertea, Sipunculida, and Annelida. in *The Reticuloendothelial System: A Comprehensive Treatise. Volume 3: Phylogeny and Ontogeny* 773 (Plenum Press, New York, 1982). doi:10.1007/978-1-4684-4166-6_4

70. Crandall, F. B., Norenburg, J. L. & Gibson, R. Gonadogenesis, embryogenesis, and unusual oocyte origin in *Notogaeanemertes folzae* Riser, 1988 (Nemertea, Hoplonemertea). in *Hydrobiologia* (1997). doi:10.1023/A:1003145519006

71. Smith, J. E. Memoirs: The Early Development of the Nemertean *Cephalothrix rufifrons*. *J. Cell Sci.* **2**, 335–381 (1935).

72. Coe, W. R. Analysis of the regenerative processes in nemerteans. *Biol. Bull.* **66**, 304–315 (1934).

73. Xing, J. & Qian, P. Y. Tower cells of the marine bryozoan *Membranipora membranacea*. *J. Morphol.* (1999). doi:10.1002/ (SICI)1097-4687 (199902)239:2<121::AID-JMOR1>3.0.CO;2-1

74. Dyrynda, P. E. J. & King, P. E. Gametogenesis in placental and non‐placental ovicellate cheilostome Bryozoa. *J. Zool.* (1983). doi:10.1111/j.1469-7998.1983.tb02810.x

75. Temereva, E. N. & Malakhov, V. V. The circulatory system of phoronid larvae. *Dokl. Biol. Sci.* **375**, 712–714 (2000).

76. Pardos, F., Roldán, C., Benito, J. & Emig, C. C. Fine Structure of the Tentacles of *Phoronis australis* Haswell (Phoronida, Lophophorata). *Acta Zool.* (1991). doi:10.1111/j.1463-6395.1991.tb00320.x

77. Malakhov, V. V. & Temereva, E. N. Embryonic Development of the Phoronid *Phoronis ijimai*. *Russ. J. Mar. Biol.* (2000). doi:10.1023/A:1009494621160

78. Temereva, E. n. & Malakhov, V. v. Embryogenesis and larval development of *Phoronopsis harmeri* Pixell, 1912 (Phoronida): Dual origin of the coelomic mesoderm. *Invertebr. Reprod. Dev.* (2007). doi:10.1080/07924259.2007.9652228

79. Rowley, A. F. & Hayward, P. J. Blood cells and coelomocytes of the inarticulate brachiopod *Lingula anatina*. *J. Zool.* (1985). doi:10.1111/j.1469-7998.1985.tb05609.x

80. Kuzmina, T. V., Temereva, E. N. & Malakhov, V. V. Ultrastructure of the lophophoral coelomic lining in the brachiopod *Hemithiris psittacea*: functional and evolutionary significance. *Zoomorphology* (2018). doi:10.1007/s00435-018-0397-8

81. Sminia, T. Phagocytic Cells in Molluscs. in *Aspects of Developmental and Comparative Immunology* (1981). doi:10.1016/b978-0-08-025922-2.50022-x

82. Saleuddin, A. S. M. & Khan, H. R. Motility of the oocyte of *Helisoma* (Mollusca). *Eur. J. Cell Biol.* (1981).

83. Saleuddin, A. S. M. & Farrell, C. L. Brain extract causes amoeboid movement in vitro in oocytes in Helix aspersa (Mollusca). *Int. J. Invertebr. Reprod.* (1983). doi:10.1080/01651269.1983.10510021

84. Bretschneider, L. H. & Raven, C. P. Structural and Topochemical Changes in the Egg Cells of *Limnaea stagnalis* L. During Oogenesis. *Netherlands J. Zool.* (1954). doi:10.1163/036551654X00113

85. Blaauw-Jansen, G. On the influence of temperature and of lithium chloride on the amoeboid mobility of unsegmented eggs of *Limnaea stagnalis* L. *Proc. K. Ned. Akad. van Wet.* **53**, 910–912 (1950).

86. Lyons, D. C., Perry, K. J., Lesoway, M. P. & Henry, J. Q. Cleavage pattern and fate map of the mesentoblast, 4d, in the gastropod *Crepidula*: a hallmark of spiralian development. *Evodevo* 21 (2012).

87. Lyons, D. C., Perry, K. J. & Henry, J. Q. Spiralian gastrulation: Germ layer formation, morphogenesis, and fate of the blastopore in the slipper snail *Crepidula fornicata*. *Evodevo* (2015). doi:10.1186/s13227-015-0019-1

88. Franchini, A. & Ottaviani, E. Repair of molluscan tissue injury: Role of PDGF and TGF-β. *Tissue Cell* (2000). doi:10.1054/tice.2000.0118

89. Malakhov, V. V. & Berezinskaya, T. L. Structure of the Circulatory System of Arrow Worms (Chaetognatha). *Dokl. Biol. Sci.* **376**, 78–80 (2001).

90. Carré, D., Djedlat, C. & Sardet, C. Formation of a large Vasa-positive germ granule and its inheritance by germ cells in the enigmatic Chaetognaths. *Development* (2002).

91. Duvert, M., Perez, Y. & Casanova, J. P. Wound healing and survival of beheaded chaetognaths. *J. Mar. Biol. Assoc. United Kingdom* (2000). doi:10.1017/S0025315400002873
