# Supplementary material for: A flagellate-to-amoeboid switch in the closest living relatives of animals
Source: eLife. 2021 Jan 15;10:e61037. doi: 10.7554/eLife.61037 (PMC7895527; doi:10.7554/eLife.61037)
Supplement: Supplementary file 2. [file elife-61037-supp2.docx]

| **Supplementary File 2. Compounds used in pharmacological assays** | | | |
| --- | --- | --- | --- |
| **Name (target)** | **Provider** | **Stock solution** | **Working concentration** |
| Blebbistatin (myosin II) | Sigma-Aldrich B0560 | 17 mM in 90% DMSO/10% H2O | 17 μM |
| Latrunculin B (F-actin) | Sigma-Aldrich L5288 | 10 mM in DMSO | 2-20 μM |
| CK-666 (Arp2/3) | Sigma-Aldrich SML0006 | 100 mM in DMSO | 10-100 μM |
| BAPTA-AM (intracellular Ca^2+^) | ThermoFisher Scientific B6769 | 32.7 mM in DMSO | 327 μM |
| Actinomycin D | Sigma-Aldrich A1410 | 20 mg/mL in DMSO | 0.1 mg/mL |
